# The Role of Nutri(epi)genomics in Achieving the Body’s Full Potential in Physical Activity

**DOI:** 10.3390/antiox9060498

**Published:** 2020-06-07

**Authors:** Irene Petracci, Rosita Gabbianelli, Laura Bordoni

**Affiliations:** 1 School of Advanced Studies, University of Camerino, 62032 Camerino, Italy; irene.petracci@unicam.it; 2 School of Pharmacy, Unit of Molecular Biology, University of Camerino, 62032 Camerino, Italy

**Keywords:** nutrigenomics, epigenetics, physical activity, epigenetic memory, nutrition, antioxidant foods

## Abstract

Physical activity represents a powerful tool to achieve optimal health. The overall activation of several molecular pathways is associated with many beneficial effects, mainly converging towards a reduced systemic inflammation. Not surprisingly, regular activity can contribute to lowering the “epigenetic age”, acting as a modulator of risk toward several diseases and enhancing longevity. Behind this, there are complex molecular mechanisms induced by exercise, which modulate gene expression, also through epigenetic modifications. The exercise-induced epigenetic imprint can be transient or permanent and contributes to the muscle memory, which allows the skeletal muscle adaptation to environmental stimuli previously encountered. Nutrition, through key macro- and micronutrients with antioxidant properties, can play an important role in supporting skeletal muscle trophism and those molecular pathways triggering the beneficial effects of physical activity. Nutrients and antioxidant food components, reversibly altering the epigenetic imprint, have a big impact on the phenotype. This assigns a role of primary importance to nutri(epi)genomics, not only in optimizing physical performance, but also in promoting long term health. The crosstalk between physical activity and nutrition represents a major environmental pressure able to shape human genotypes and phenotypes, thus, choosing the right combination of lifestyle factors ensures health and longevity.

## 1. Introduction

The World Health Organization (WHO) defines “physical activity” as any bodily movement produced by skeletal muscles that requires energy expenditure [1]. All activities done as part of playing, working, active transportation, house chores or exercise (planned, structured, and repetitive bodily movement) are covered by this definition. Over the last decades, increasing interest from the scientific community has been conveyed into the effects that an active or inactive lifestyle may exert on human health. The evidence generally suggests that physical activity and exercise positively impact human health, promoting biological and physiological changes, beneficial for optimal health and well-being. In this view, physical activity represents a powerful tool to prevent, improve or even revert several chronic medical conditions, from metabolic disorders to autoimmune diseases, cardiovascular disease and cancer [2]. Estimates by the WHO attribute approximately 3.2 million deaths per year to physical inactivity, making it the fourth leading risk factor for global mortality [3]. According to current international guidelines from the WHO, healthy adults (aged 18–64) should do at least 150 min of moderate-intensity aerobic physical activity throughout the week [1]. Physical exercise allows the simultaneous and integrated work of pulmonary, respiratory, skeletal muscle and cardiovascular systems. Among these, the cardiovascular system plays a central role, because the increase of the heart rate and changes in the blood flow are necessary to meet oxygen demand, consequent to the aerobic work. Epidemiological studies have proved that regular exercise can prevent or mitigate numerous non-communicable diseases, such as cardiovascular disease, type 2 diabetes and cancer [4]. This review aims to emphasize the role of nutrition in influencing skeletal muscle activity and its adaptive response to exercise. In this regard, we want to highlight how nutrition, balanced in its macro and micro-nutrient content and rich in natural antioxidants, can epigenetically fine-tune skeletal muscle gene expression and metabolite production, as well as contribute to the maintenance of oxidative homeostasis through the regulation of mitochondrial function.

## 2. Physical Activity: The Molecular Side of the Coin

Extensive gene expression changes are induced by physical activity in different organs for several important aims. All physiological systems of the body are involved in these changes, in order to maintain the overall body homeostasis, such that contracting skeletal muscles are continuously supplied with energy and oxygen, metabolic waste products are properly removed, and vital body functions are not compromised. In skeletal muscle, contractile myofibers respond to external stimuli, like physical activity, undergoing molecular and physical remodeling as a form of adaptation. This adaptation requires a complex intracellular signal transduction that activates numerous downstream pathways, ultimately culminating with the myofiber remodelling through changes in gene expression.

A clear adaptation to exercise is the variation in skeletal muscle size. Repeated bouts of muscle loading lead to muscle hypertrophy (increased muscle mass), through the activation of serum response element 1, a cis-acting regulatory element that homodimerizes to serum response factor [5] and activates α-actin promoter transcription, leading to an increase in contractile protein availability in response to overload conditions [6]. Increased insulin-growth factor-1 (IGF-1) isoforms within muscle fibers, upon muscle loading, has been documented [7]. IGF-1, via phosphatidylinositol-3-kinase [8], activates the protein kinase B (also known as Akt), leading to muscle hypertrophy. Moreover, activation of other pathways results in enhanced skeletal mass. One of these is calcineurin, a Ca^2+^-calmodulin-dependent phosphatase that, via dephosphorylation of the nuclear factor of activated T cells, induces myofiber hypertrophy [9]. In overloaded muscles, calcineurin is most likely activated via the intracellular increase of calcium concentration upon muscle fiber activation and increases in IGF [9].

With physical exercise, also the oxidative properties of skeletal muscles change towards the increase in muscle oxidative capacity, thanks to mitochondria biogenesis and upregulated mitochondrial protein expression. Exercise stimulates mitochondrial biogenesis (measured through cytochrome c protein expression levels), inducing 5′AMP-activated protein kinase (AMPK) activity [10]. AMPK activates the transcription of several molecules, including glucose transporter 4 (GLUT-4), hexokinase, uncoupling protein 3, some mitochondrial oxidative enzymes, and nuclear respiratory factor 1 (NRF-1), a transcription factor that, binding to the delta-aminolevulinic acid (ALA) synthase and mitochondrial transcription factor (mTFA) gene promoters, upregulates cytochrome c (Cyt c) protein expression [11]. The increase in mitochondrial copy number seems to be an adaptation to exercise, to limit homeostasis perturbation. In fact, in the case of repeated exercise, muscles preferentially oxidize fatty acids (instead of glycogen reservoir), as evidenced by increased β-oxidation enzymes [12] and decreased inorganic phosphate ADP, AMP and lactate concentrations [13]. Norepinephrine and epinephrine, released upon physical activity, bind to lipolysis-inducing β-adrenoceptors (β-ARs), leading to an increase in intracellular cyclic AMP (cAMP) concentrations and to the activation of protein kinase A (PKA), culminating with lipolysis [14]. Exercise-induced adipose tissue lipolysis increases by 2.5-fold from rest during low intensity activity, to reach a plateau at higher exercise intensities [15]. As a result of fatty acids’ oxidation, hypoglycaemia-induced fatigue risk is prevented and a longer muscle endurance is achieved [16]. After all, the increased request of ATP, obtained by the oxidative phosphorylation pathway in mitochondria, is associated with the monoelectronic reduction of oxygen to water and to the release of reactive oxygen species (ROS), which can be neutralized by the endogenous (i.e., antioxidant enzymes) and the exogenous (i.e., antioxidant foods) redox system. 

The AMPK signaling pathway is also implicated in the glucose uptake by skeletal muscles, without insulin involvement. In fact, as already mentioned, increased AMPK upon contraction leads to the upregulation of GLUT4 and hexokinase, which favor glucose uptake, and mitochondrial oxidative enzymes for aerobic respiration [17]. In addition, glucose uptake is also favored by the increase in sarcoplasm Ca^2+^ concentrations, in an AMPK-independent way, through the activation of myocyte enhancer transcription factors 2A and 2D (MEF2A and MEF2D) [18]. These molecular pathways partially explain the important role of exercise in preventing or reducing type 2 diabetes. This data are also confirmed by a study of Heath et al., who showed that, in healthy subjects, insulin resistance increases within 5–10 days after the interruption of physical activity [19].

The molecular and physiological adaptations encountered by trained muscles represent one of the main differences with the exercise-deficient skeletal muscle, which instead, for equal training duration and intensity, undergoes a remarkable disruption of homeostatic balance [20]. Exercise training is able to modulate the cardiovascular system physiology, improving vessel health and cardiac activity. Exercise leads to the up-regulation of endothelial nitric oxide synthase (ecNOS) mRNA, with resulting increases in the powerful vasodilator NO [21] and superoxide dismutase (ecSOD) [22]. The latter is essential for its free radical scavenging activity: SOD converts superoxide anion radicals into hydrogen peroxide, thereby preventing the formation of toxic metabolites, such as peroxynitrite, which will degrade NO. Via NO, physical exercise leads to increased coronary diameter, enhanced coronary flow and reduced shear stress, moreover, through the inhibition of atherogenic factors (platelet aggregation, leucocytes adhesion and smooth muscle cell proliferation), it contributes to the prevention of atherosclerosis and myocardic ischemia [23]. Another form of adaptation to exercise is the so called “athletic heart”, which is a physiological left cardiac hypertrophy, characterized by increased cardiac reserve of up to 500–600% [24]. The molecular mechanisms underlying the increased cardiac volume can be addressed to the activation of the calcineurin signaling cascade [25], triggered by physical activity upon release of IGF-1 and noradrenaline [26]. 

Beneficial effects of regular physical activity have been observed in particular against diseases associated with systemic inflammation. Indeed, modulation of gene expression is also essential in regulating the release of anti-inflammatory “myokines” from the muscles (IL-6, IL-1ra, IL-10, TNF-R) which, in turn, lead to the downregulation of pro-inflammatory cytokines (TNF-α, IL-1, CRP) [27]. Contracting skeletal muscle secretes “myokines” with beneficial effects on peripheral target organs and deserves the designation of a full-fledged endocrine organ [28]. Endocrine functions attributed to skeletal muscle are involved in body weight regulation [29], low-grade inflammation [30] and significant improvements in glucose tolerance and insulin sensitivity [31], as well as better cognitive function, with reduction of stress, anxiety and depression [32], and tumor-growth suppression [2]. Anti-cancer properties of physical activity have been ascribed to the stimulation of the immune system upon exercise, leading to an increase in the circulating immune cells count. During training, in fact, catecholamines (adrenaline and noradrenaline), which are massively released by the adrenal glands, besides being responsible for increased heart rate, blood flow and circulating glucose levels, cause the mobilization of NK cells, expressing the catecholamines β-adrenergic receptors. Redistribution of NK cells is also due to the release of IL-6, upon acute physical exercise, in an intensity-dependent manner (NK cells express the IL-6 receptor complex) [4]. Considering the ability of NK cells to kill cancer cells [33], physical activity could also represent an important adjuvant in anticancer therapies. Recent studies also attribute to exercise the ability to increase the expression levels of p53, a well-known tumor suppressor protein [34], confirming, once again, that physical activity may counteract tumor development or slow down tumor progression [35,36]. 

Nevertheless, physical exercise may hide some drawbacks. Since the 1970s, several studies have reported that physical exercise promotes cellular oxidative stress [37,38], which is defined as the “imbalance between oxidants and antioxidants in favor of the former, leading to a disruption of redox signalling and control and/or molecular damage” [39]. In oxidative conditions, ROS and reactive nitrogen species (RNS), often referred to as RONS or “free radicals”, exceed the body’s antioxidant defense and promote inflammation. Free radicals are the physiological bio-products of muscle fiber contraction, in response to increased oxygen demand [40], increased muscle temperature and CO_2_ levels, and lowered pH [41]. Being chemically unstable (because of an unpaired electron), these free radicals tend to react with and oxidize redox-sensitive targets in the cell (lipid, protein and DNA), favoring the onset of oxidative damage-linked pathologies, including cancer [42], diabetes and neurodegeneration [43]. Paradoxically, physical exercise, which is associated with many health benefits and reduced risks for the above mentioned pathologies, acts as a source of oxidative damage in the first place. The effect of exercise on redox balance is complex and affected by factors like age, gender, training level, intensity and duration of exercise. The truth is that physical activity plays a pivotal role in regulating the balance between reactive species production and the body’s antioxidant mechanisms. Free radicals are the primary cause of exercise-induced disturbances in muscle redox balance, but skeletal muscle fibers have enzymatic and non-enzymatic (glutathione, vitamin E, vitamin C, lipoic acid, carotenoids, uric acid, bilirubin and ubiquinone) defense mechanisms to limit the risk of oxidative damage. Interestingly, regular and moderate exercise is beneficial for oxidative stress and health, while acute exercise or endurance training causes a transient elevation of free radical production, but, at the same time, provides the necessary stimulus for up regulation of endogenous antioxidant defenses [44]. Indeed, contraction-induced ROS production seems to trigger a signalling cascade that leads to muscle adaptation, in terms of muscle gene expression modulation and performance optimization. The increase of ROS activates the nuclear factor erythroid 2-related factor 2 (Nrf2), known as antioxidant cytoprotector master regulator. Nrf2 dissociates from its inhibitor Keap1, and translocates in the nucleus, where it binds to specific a DNA sequence known as the antioxidant response element (ARE, 5′-TGACNNNGC-3′), and activates genes associated with the antioxidant and detoxifying response. Phase-II antioxidant enzymes, glutathione synthesis, ROS elimination and NADPH synthesis are stimulated upon Nrf2 signal [45]. 

Furthermore, it was demonstrated that exercise leads to the activation of mitogen-activated protein kinases (MAPKs: p38, ERK 1 and ERK 2), which in turn activate the nuclear factor kappa light-chain-enhancer of activated B cells (NF-κB), resulting in the expression of antioxidant enzymes (like SOD), important for the defense against ROS and adaptation to exercise [46]. However, a massive ROS production, following an acute burst of intense exercise, causes contractile dysfunction (resulting from muscle protein oxidation) [47] and muscular atrophy (resulting from increased muscle protein degradation) [48], with a consequent reduction in muscle force and increased muscle fatigue [49]. Elevated ROS levels can also alter myofilament structure and function [50], compromising their calcium sensitivity [51], thus contributing to muscle fatigue. 

## 3. Epigenetics Mediates Molecular Effects of Physical Activity

Remarkably, recent studies have demonstrated that physical activity can regulate gene expression also through epigenetic changes. The term “epigenetics”, literally “above the genome”, refers to those processes that modulate gene expression without entailing a change in DNA nucleotide sequence (mutation) [52]. Epigenetic changes consist of reversible “tags”, mitotically and/or meiotically heritable, that can be applied to DNA or histone proteins, contributing to the overall chromatin structure. Thus, epigenetics changes the phenotype, in absence of a corresponding genotypic variation. This explain why, although all cells of an organism share an identical genetic code, each cell type has a distinctive gene expression pattern, which is, indeed, driven by a specific epigenetic signature. 

Epigenetic modifications can occur directly on the DNA molecule via methylation, or at the level of histone proteins (prone to methylation, acetylation, phosphorylation, ubiquitination, SUMOylation, ADP-ribosylation and citrullination) [53]. Both types of modifications affect gene transcription and expression. DNA methylation is controlled by DNA methyltransferases (DNMTs) and demethylases, and mainly occurs on CpG dinucleotide pairs creating 5-methylcytosine (5mC). Cytosine methylation within promoters can mask transcription factor binding sites on DNA [54], or facilitates methyl group-binding transcriptional repressors [55], leading to gene suppression [56]. However, if it occurs at intragenic sites, DNA methylation can have a variable impact on gene transcription. Compared to DNA methylation, histone modifications are more complex and, as the name suggests, they occur on histones. Histone proteins are the main components of chromatin, responsible for the packaging of DNA into structural units called nucleosomes. They consist of many amino acids with basic side chains (in particular lysine and arginine residues) which are the main target of epigenetic modifications. These latter are post-translational alterations, whose effect on gene transcription depends on either the type of modification or the target amino acid on histone tails. Histone modifications can promote gene silencing/activation, directly modulating the chromatin structure. Normally, an “open” chromatin structure (euchromatin) facilitates accessibility to promoters of transcriptional machinery, and is associated with transcriptional activation and gene expression, while a “closed” chromatin structure (heterochromatin) is associated with transcriptional repression and gene silencing [57]. Generally, histone acetylation favors a looser chromatin structure and leads to active transcription. In fact, acetylation removing the positive charge of histones decreases their interaction with the negatively charged phosphate group of DNA, and this translates into a more relaxed chromatin structure, which enhances gene transcription. On the other hand, histone methylation can have both repressing and activating effects. 

Within the bounds of epigenetic regulation, microRNAs (miRNAs) shall also be mentioned. miRNAs are short non-coding RNA molecules, 18–24 nucleotides in length, which act as important post-transcriptional regulators of messenger RNA (mRNA), thus having a key role in RNA silencing and in the control of gene expression. They prevent mRNA’s translation into proteins by binding to the complementary region in the 3′UTR (untranslated regions) of mRNA molecules [58]. On the basis of this base-pairing, their silencing effect can be either transient (temporary translational downregulation) or permanent, with degradation of the bound target mRNA [59]. They have been found in viruses, plants and animals, and about 2603 miRNA species have been identified in humans so far [60]. In mammals, miRNAs have been found in many cell types and are responsible for the downregulation of about 60% of human and other mammalian genes [61]. However, it has been proved that miRNAs also exist in a stable, cell-free circulating form, and approximately 300 species have been found in plasma, saliva, urine, serum and other extracellular fluids [60,62,63]. Circulating miRNAs may be useful as clinical biomarkers for numerous pathologies, such as, among others, obesity-related diseases (i.e., type 2 diabetes or cardiovascular disease) [64,65]. Interestingly, a modest pool of miRNAs was found in mitochondria (mitomiRs), where they are involved in the control of mitochondrial homeostasis and may be linked to inflammation processes [66]. Mainly nuclear-encoded, but also of mitochondrial origin, mitomiRs have been hypothesized to fine-tune proteins regulating cell survival (mainly Bcl-2 family members) balancing anti- and proapoptotic signals and controlling ROS production, mitochondrial fusion/fission, and mitophagy [67]. miRNAs production is highly dynamic and it has been proven that, among other factors, physical activity can influence the expression of more than 100 miRNAs [68]. Some of them are known to be implied in specific cancers [69,70], metabolic [71] and cardiovascular diseases [72].

**Figure 1:**
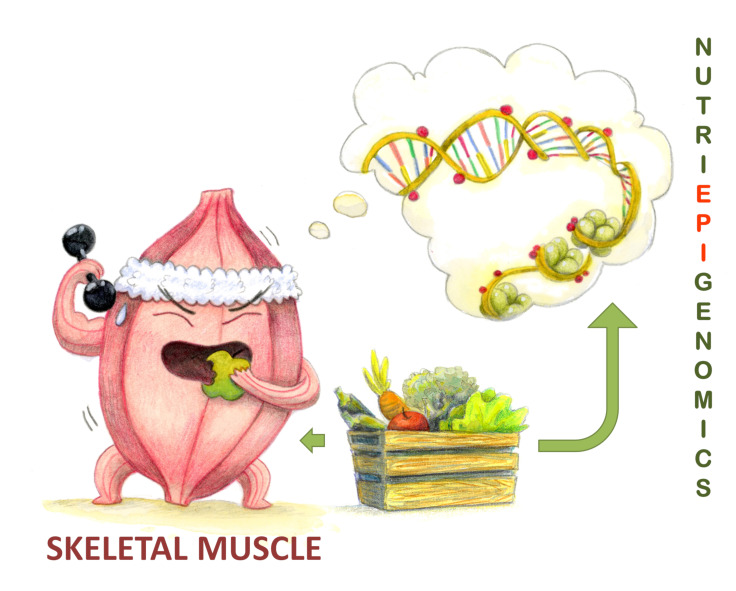
Nutritional status and epigenetic memory affect muscle response to physical activity. Nutri(epi)genomics modulates physical performance affecting skeletal muscle trophism and its response to external stimuli, by modulating numerous molecular pathways through epigenetic regulations of the involved genes.

It is through DNA methylation, histone post-translational modifications and miRNA-induced gene expression regulation, that the individual epigenetic imprint, referred to as “epigenome”, governs the activation or silencing of genes on DNA [73], contributing to the non-genetic variation of the phenotype. Epigenetic modifications naturally occur during life, as part of the physiological cellular differentiation process (from pluripotent stem cells to fully committed cells that form specialized tissues and organs) or through genomic imprinting. Moreover, the epigenome is highly flexible and dynamic, as well as extremely susceptible to biological and environmental factors, such as aging and lifestyle factors, including nutrition, antioxidant foods, stress, toxins, smoke and, last but not least, physical exercise. In fact, physical activity has recently gained popularity for its contribution to health and well-being, also through epigenetic modulation of gene expression. Epigenetic modifications induced by physical activity can be transient, such as those occurring after a high-intensity aerobic exercise, but they can be also stable if exercise is part of a lifestyle. In this latter case, the epigenetic changes build the skeletal muscle memory, which is defined as the ability of skeletal muscle to respond or adapt differently to environmental stimuli which have been previously encountered [74]. Skeletal muscle can be programmed and can “remember” several stimuli faced earlier in life. In particular, skeletal muscle can retain information after a certain stimulus/stress, in order to be primed for future plasticity, in case it should re-encounter the same stimulus later in life. This behavior is underpinned by distinct molecular mechanisms, which epigenetics is part of (Figure 1). In the context of physical activity, after injury or the off-season, this “epi”- memory allows the muscle to respond more quickly, but also morphologically and functionally differently, to training (stimulus) that has already been experienced in the past [74].

Upon exercise, hypomethylation of metabolic gene promoters has been documented. To mention some, the peroxisome proliferator-activated receptor gamma coactivator 1-alpha (PGC-1α), the peroxisome proliferator-activated receptor delta (PPARD), the mitochondrial transcription factor A (TFAM) and the myocyte enhancer factor 2A (MEF2A); interestingly, many of these genes are hypermethylated in diabetes [75], and this can explain why regular exercise inversely correlates with metabolic diseases [76]. A study conducted on sedentary men showed how repeated sessions of aerobic work over six months led to global metabolic changes (like reduced waist/hip ratio, increased HDL levels and reduced blood pressure), accompanied by changes in DNA methylation profile in adipose tissue genes linked to obesity and diabetes [77]. As a further confirmation, in a healthy people cohort, physical inactivity (as a lifestyle or following injuries) altered their metabolic state, inducing insulin resistance, with widespread changes in oxidative metabolism [78]. These changes were ascribed to an altered epigenetic signature with consequential gene expression modulation (i.e., increased methylation of the promoter region of the PGC-1α gene, which is important for mitochondrial functions and oxidative metabolism). It is worth noting that oxidative gene expression wasn’t restored within 4 weeks of retraining, underscoring the potential long-lasting epigenetic response to physical inactivity [78]. Epigenetic changes induced by exercise were also examined in subjects with heart failure [79]. The major contribution to this condition is chronic sterile inflammation, molecularly characterized by the formation of the inflammasome, a protein complex that activates inflammatory cytokines that promote cardiac hypertrophy and myocardial apoptosis [80,81]. The apoptosis-associated speck-like protein, containing a caspase recruitment domain (ASC), is an important component of the inflammasome, thus a key mediator of the cytosol-type inflammatory signalling pathway, and its expression is epigenetically controlled by DNA methylation. In general, low ASC methylation is linked to poor prognosis in heart failure. Interestingly, a 3-month exercise intervention in subjects with heart failure was able to increase ASC methylation levels. The increase in ASC methylation after exercise was associated with a decrease in ASC mRNA expression and in the inflammatory cytokine IL-1β, suggesting a role for physical activity in reducing inflammation, thereby preventing diseases linked to a low-grade chronic inflammation [79].

Furthermore, it was observed that, upon endurance training, the methylation profile of hundreds of genes in the vastus lateralis was modified, with changes in intracellular responses involving structural remodeling or carbohydrate metabolism [82,83]. Non-endurance strength training also triggered changes in leukocyte DNA methylation patterns, modulating anabolic growth factor expression, most likely as an epigenetic adaptation to face muscle damage [84]. Interestingly, an intensity-dependent correlation between exercise and epigenetic response seems to exist. In this regard, high-intensity exercise, that is 80% maximal aerobic capacity (VO_2max_), was linked to promoter hypomethylation of several oxidative genes, fostering oxidative metabolism [75]. On the other hand, low-intensity exercise (40% VO_2max_) didn’t seem to elicit the same epigenetic response, unless over long periods [85]. In humans, the epigenetic response to physical activity has mainly been studied on skeletal muscle and adipose tissue because of their central role in exercise or chronic disease onset (like diabetes) and because of their accessibility via biopsy. However, studies on rat and mouse models revealed an interesting role of aerobic exercise in also promoting epigenetic changes in the hippocampal region [86,87]. In this regard, hypomethylation of brain-derived neutrophic factor (BDNF) gene promoter, which is crucial for learning, memory function and mood, has been documented [88,89]. To date, one human study attributes a possible role in modulating neurodegeneration progression to exercise, through the silencing of genes which exert pivotal roles in schizophrenia [90]. 

In recent decades, several studies have been addressed to understand the correlation between changes in DNA methylation profile and aging. Since “chronological age” is not always predictive of the aging process, it was proposed to identify markers of “biological age”, in other words, multi-tissue biomarkers that can serve to accurately and rapidly predict how the functionality of body organs changes with age [91]. It has been documented that the methylation status of many CpG sites varies during a lifetime [92,93], and an overall decrease in DNA methylation, along with an increased inter-individual variability of DNA methylation, have been observed with aging [93,94,95,96]. Horvath, through the analyses of publicly available DNA methylation data sets, was the first to develop an accurate model of human aging, which estimates the biological age for multiple tissues and organs through DNA methylation [97]. Despites general patterns of DNA methylation individually changing during aging, it was observed that the methylation status at 353 specific CpG sites was directly associated with biological age and it could be used to predict epigenetic age (DNAm age), which is not only a reflection of the person’s chronological age, but also of the DNA source biological age [97]. A positive “age acceleration”, in other words, the discrepancy between DNAm age and chronological age, with the former being greater than the latter, correlates with several health issues and predicts all-cause mortality [98,99]. Interestingly, comparative reviews concluded that the epigenetic clock is the most reliable molecular estimator of biological age among other potential biological age estimators (such as telomere length, transcriptomic-based, proteomic-based and metabolomic-based biomarkers) [100,101]. Epigenetic age differences among individuals with the same chronological age could be extremely useful to study the impact of endogenous or exogenous stressors on the ageing process. Furthermore, the reversibility of epigenetic changes highlights the most exciting feature of DNAm biomarkers: choosing the right combination of lifestyle factors, biological aging rates can be controlled, in favor of a balance between chronological and biological ages. Physical activity fits perfectly into this context and, as well as other behavioral lifestyle factors including diet and alcohol consumption, it might represent a promising anti-ageing intervention. In fact, large epidemiological studies have proven that regular exercise can contribute to lower the “epigenetic age,” reducing metabolic disease risk and enhancing longevity. Researchers noticed that subjects displaying a low epigenetic age not only were engaged in physical activity but also had healthier dietary habits (consuming moderate alcohol and more fruits, vegetables, fish or poultry) [102]. An increased age acceleration was found in the liver of obese subjects [103]. The association between body mass index (BMI) and increased age acceleration was also reported by other studies [102,104]. All together, these findings support the theory that physical activity, promoting body weight control, might be a key factor in slowing age acceleration, as is also confirmed by a study on blood cell methylation status conducted on the Swedish population [105]. These studies provide a first evidence that exercise, working in partnership with diet, is a powerful modulator of “epigenetic age” and contributes to long-lasting effects on overall health, protecting from metabolic diseases and favoring longevity. 

Not only DNA methylation, but also histone modifications, are involved in the regulation of the responses to physical activity. According to several studies, physical exercise promotes histone acetylation, in particular at H3 and H4 level, with a positive impact on several diseases like cancer [106], muscle wasting [107] and behavioral disorders [90], through the inhibition of specific genes related to them.

Finally, through the modulation of miRNA’s expression in skeletal muscle, physical activity can influence mitochondrial metabolism, inflammation, muscle recovery and hypertrophy [108]. In this context, the identification of miRNA’s expression pattern induced by physical exercise could be an important tool to monitor physical fatigue, recovery and physical performance capacity [109]. Circulating miRNAs also have promising applications as biomarkers to reveal the use of performance-enhancing drugs [110]. As already mentioned, skeletal muscle is a plastic organ, able to adapt to a variety of external stimuli (such as exercise, inactivity or nutrition), thus modifying its phenotype. In this context, miRNAs, either tissue-specific (commonly referred to as myomiRs, including miR-1, miR-133, miR-206, miR-208, miR-486 and miR-499) or not, play a key role in the modulation of muscle tissue myogenesis, development and function [111,112]. MyomiRs expression pattern is highly affected by external stimuli, like exercise or inactivity. The first studies were conducted on skeletal muscle biopsies and they revealed differential expression of muscle-specific miRNAs in response to endurance training [113,114,115]. More recently, due to the discovery of circulating miRNAs body fluids and the advent of new technologies for their detection and quantification, several studies have focused on the influence of physical exercise on plasma and serum circulating miRNA levels, and they revealed a differential expression pattern according to the type of exercise [116,117,118]. In fact, aerobic endurance and strength or resistance-type exercise have the ability to evoke opposite responses, by activating opposite signalling pathways and differential gene expression finely regulated by miRNAs. While endurance exercise activates mitochondrial biogenesis, muscle fast-to-slow twitch fiber-type transformation and substrate metabolism, strength exercise mainly increases protein synthesis, promoting muscle hypertrophy [119]. The differential expression profile of circulating miRNAs in serum or plasma may be due to adaptation to specific exercise types, in fact their expression varied depending on endurance versus strength training or acute versus chronic stimulation. A study revealed a possible role for exercise to modulate insulin sensitivity through miRNAs. In this regard, the miR-378 family, which is increased in skeletal muscle after a bout of interval training, may regulate insulin sensitivity by the posttranscriptional regulation of PPAR genes [120]. Instead, miR494, whose levels decrease upon 7 days of endurance exercise, inhibits mitochondrial biogenesis by targeting either the mitochondrial transcription factor A (TFAM) or Forkhead Box J3 (FOXJ3) genes [121]. 

Despite the fact that there are still many challenges associated with the detection of circulating miRNAs (the lack of standardized protocols to minimize protocol-based bias), it is clear that several tissue-specific miRNAs are released into circulation during and after exercise, as a response to the physiological stimulus. Moreover, their expression pattern appears to be influenced by and specific to exercise type and intensity.

Throughout all of these molecular mechanisms, it appears that mitochondria are major players in regulating the response to physical activity. Indeed, mitochondria are essential for cell survival, being involved in adenosine triphosphate (ATP) production, metal homeostasis, regulation of cellular metabolism and respiration. Within the organelle matrix, multiple copies of circular, double-stranded DNA (mtDNA) are present and they are independent of the nuclear genome (nDNA) [122], but in a strict cross-talk with it [123]. Exercise induces rapid intracellular signals, through an intricate interplay between the nuclear and mitochondrial genomes, culminating in the overexpression of several mitochondrial proteins. Among these, the peroxisome proliferator-activated receptor gamma coactivator 1-α (PGC-1α) increased expression upon exercise and has been observed in skeletal muscle. Since PGC-1α regulates mitochondrial biogenesis, upregulation of PGC-1α in muscle cells during exercise results in an increased mitochondrial copy number [124]. The reasons behind the stimulation of mitochondrial biogenesis with exercise are quite intuitive, given that skeletal muscle requires energy in the form of ATP, and thus, is strictly dependent on mitochondrial oxidative phosphorylation [125]. However, ATP production is accompanied by the release of free radicals which, when overwhelming the cell antioxidant defenses, cause oxidative damage to mitochondria, eventually leading to cell death. Thus, a correct balance between mitochondrial biogenesis and mitophagy (that is the selective removal of damaged mitochondria) is critical for maintaining mitochondrial quality control and cell viability under basal and stress conditions [126]. This is particularly important in aging, when mitochondria are more prone to damage, because of imbalanced ROS production-antioxidant defenses and accumulated mutations on either genomic or mitochondrial DNAs [127,128]. Evidence suggests a mitochondrial role in the aetiology of sarcopenia (the age-related loss of muscle mass and function) because of reduced physical exercise, sedentary inactivity and defective mitochondrial quality controls experienced with aging [129]. 

Remarkably, several studies demonstrated that, like nuclear DNA, mtDNA can also be targeted by epigenetic modifications, especially methylation at the level of cytosine residues [130]. mtDNA methylation seems to play important roles in mtDNA replication and gene expression [131]. Interestingly, it appears that any cytosine nucleoside within mtDNA can be methylated, thus methylation is not restricted to CG dinucleotides [132]. Even though studies are still ongoing, it emerged that mitochondrial dysfunction in the form of altered gene expression and ATP production, due to epigenetic changes, may contribute to several conditions, including altered metabolism, neurodegenerative disorders, metabolic and cardiovascular diseases [133,134,135,136] and cancer [137,138]. In addition to mitochondrial epigenetics [122], mtDNA copy number can also play a significant role in the onset of numerous health and disease conditions [139,140], and physical exercise can affect also this parameter [141]. 

All together, these findings support the important role of physical activity in promoting health. Long-term exposure to exercise can promote health and longevity through epigenetic adaptations, which may be mitotically transmitted to new cells during tissue growth and repair, and eventually yield systemic changes in an organism. More interestingly, even though studies are still ongoing, these exercise-induced epigenetic changes may be passed on to the next generations (from mother to fetus and female fetal gametes) [142]. It is still not clear whether exercise training alone influences the epigenomes and health status of progeny. However, recent studies suggest that exercise may lead to intergenerational inheritance of specific traits. For example, paternal obesity was associated with transmission of metabolic syndrome risk factors to the offspring: a high-fat diet caused aberrant expression of X-chromosome-associated sperm miRNAs (miR-503, miR-542-3p and miR-465b-5p), involved in cell cycle regulation, apoptosis and embryo development pathways. Interestingly, 8 weeks of preconceptional paternal exercise normalized body weight, glucose intolerance, plasma leptin and C reactive protein concentration, which were altered on a fat-diet regimen. Thus, in this case, exercise proved to be effective in preventing the harmful epigenetic inheritance of metabolic disease [143]. On the other hand, the maternal influences of exercise on the inheritance of health and disease risk are more established. Several studies on rodents, on maternal exercise training during gestation, revealed that physical activity had a positive impact on cognition and behaviors in the progeny (improved memory and learning, reduced stress and fear), due to increased brain-derived neurotrophic factor (BDNF) expression and neurogenesis. Interestingly, these positive adaptations were also extended to adulthood [144,145]. Even though further data on transgenerational inheritance are needed, these evidences prove that both maternal and paternal exercise may be effective to counteract the harmful, transmittable epigenetic markers of metabolic or cognitive diseases, guaranteeing a higher quality life to the subsequent generation.

It is certain that physical activity represents an environmental pressure able to shape human genotype and phenotype, finely tuning specific intracellular signaling pathways to modulate gene expression, being a very powerful ally to achieve physical and mental well-being. In this complex picture, nutrition can play an important role in supporting the essential molecular pathways that triggers the beneficial effects of physical activity and can help to suppress those whose activation should be moderated.

## 4. The Role of Nutrition in Supporting Molecular Changes Induced by Physical Activity

### 4.1. Nutrition for Muscle Activity, Sustainment and Recovery: Macro- and Micro-nutrients

Diet plays a crucial role in mediating skeletal muscle adaptations, which in turn depend on the type, intensity and duration of exercise. In fact, endurance training mainly fosters skeletal muscle oxygen utilization capacity, by increasing mitochondrial biogenesis and capillary density [146,147,148]. Resistance exercise, instead, enhances myofibrillar volume (mainly in type II fibers), thus promoting skeletal muscle hypertrophy and strength [149,150]. Through the supply and timing of key macro- and micronutrients, nutrition plays a big part in optimizing exercise performance, metabolic adaptations and recovery processes. A balanced diet, providing either adequate macronutrients (proteins, carbohydrates, fats) or micronutrients through a variety of foods, should be the main goal of all athletes and people doing physical activity in general. Nutrient requirements are usually expressed as grams per kilogram (g/kg) of body weight (BW).

Macronutrients comprehend three main classes: proteins, carbohydrates, and fats. Proteins are essential for many biological processes. Despite being the fundamental constituents of muscles, tendons and other soft tissues, they have also important functions as enzymes, hormones, and neurotransmitters. Carbohydrates, despite often being linked to obesity and gut inflammation, are the major energy source used by both the central nervous system and body muscles. Carbohydrates are not all created equal, thus whole grains, fruits, vegetables, and legumes have to be preferred to refined sugars or highly processed carbohydrate sources. Fats are also essential for many vital processes in the body, being responsible for the integrity of cell membrane structure, the absorption of fat-soluble vitamins (A,D,E,K), brain health, hormone production and, ultimately, they are an energy source for muscle metabolism.

Nutritional requirements vary between endurance and resistance sports because they depend on the activity’s energy demands, thus diet should be balanced according to the exercise goal, such as gaining muscle or losing fat while maintaining lean mass [151]. According to the American College of Sports Medicine (ACSM), “athletes need to consume adequate energy during periods of high intensity and/or long duration training to maintain body weight and health and to maximize training effects” [152]. 

In the context of physical activity, healthy nutrient sources are as important as the timing of their intake in relation to exercise. Nutrient timing refers to the consumption of some macronutrients, mainly carbohydrates and proteins, in and around a workout performance, in order to optimize exercise-induced muscular adaptations and promote damaged tissue repair [141]. Not only professional athletes, but also non-athletic subjects can derive benefits from this approach. 

While during moderate intensity exercise (30–65% of VO_2_ peak), fat is the main energy source, for high intensity activities (70% VO_2max_), blood glucose and glycogen stores are the first sources of energy used by skeletal muscle. For this reason, as glycogen level drops and ATP is not enough to sustain muscle contraction, exercise output is affected [153], with the parallel increase of tissue breakdown rates [154]. For this reason, an adequate intake of carbohydrates before a workout is fundamental to provide the organism with good glycogen stores that will ensure optimized performance, especially for resistance training. Recommended daily intakes of carbohydrate are 5–12 g/kg/day, with the upper range reserved to those subjects training at moderate to high intensities (70% VO_2max_) [155,156]. Carbohydrate loading benefits (rapid increase and maximization of muscle glycogen stores) are currently undiscussed, even though there could be sex differences, thus female athletes may deplete endogenous glycogen stores to different extents [157,158]. The rate at which muscle glycogen is consumed varies primarily based on the intensity of physical activity: the greater the intensity, the more muscle glycogen is degraded. However, the glycogen store of active muscle cells cannot fall further than 10% of initial values, even after intense and prolonged exercise [159], thus, replenishment of the glycogen reservoir is essential to maintain performance [160]. Interestingly, glycogen is distributed in three distinct pools across the muscle tissue (subsarcolemmal, intermyofibrillar and intramyofibrillar glycogen), and studies have revealed that, during exercise, a larger relative utilization of intramyofibrillar glycogen occurs [161,162]. Depletion of muscle glycogen drives its own resynthesis [163], which occurs through gluconeogenesis or via lactate, particularly in the case of high-intensity exercise [164,165]. This is possible because exercise-induced glycogen depletion activates glycogen synthase and increases insulin sensitivity and permeability of the muscle cell membrane to glucose [166,167]. However, post-workout carbohydrate intake is recommended to replenish muscle glycogen storage and minimize the symptoms of muscle fatigue. As mentioned before, complex carbohydrates with low glycemic index have to generally be preferred to refined and simple ones [168], except when taken immediately after a workout, when the glucose uptake from muscles is stimulated and high glycemic index carbohydrates can be tolerated [169]. Lately, a novel approach to optimize skeletal muscle adaptation strategies has been proposed. In fact, several studies have reported that, at least for endurance exercise, low glycogen availability may improve oxidative capacity and strategically enhance the response in exercise-induced signalling [170,171,172,173].

Given the essential role of proteins for the body (i.e., production of antibodies, enzymes, structural components, transporters and messengers), adequate amino acid intake is required for optimal physical and physiological function. The addition of proteins (0.2–0.5 g/kg/h) to carbohydrates (<1.2 g/kg/h) has proven to be beneficial in increasing glycogen resynthesis, minimizing muscle damage, promoting hormone balance and accelerating recovery from intense exercise [155,174,175]. In particular, the addition of 6–20 g of essential amino acids (amino acids that cannot be synthesized by humans and must therefore be introduced by diet) to at least 30–40 g high glycaemic carbohydrates, immediately or within three hours post exercise, seems to stimulate muscle protein synthesis [168]. In fact, after protein ingestion, insulin secretion is activated, as well as anabolic signalling pathways that stimulate amino acid incorporation into muscle proteins [176]. Resistance training demands higher protein intakes to support muscle protein synthesis, reduce muscle protein breakdown and repair muscle damage [177]. Endurance exercise, instead, demands protein intake to counteract the increase of protein oxidation [177]. Among the recommended sources of protein, there are lean meats and fish, cottage cheese, eggs, plain Greek yogurt or protein shakes. If on a vegan diet, important protein sources are lentils, tempeh, chickpeas, black beans, quinoa, almonds, and vegan protein shakes. However, postprandial muscle protein accretion is limited and cannot be increased by eating more protein. In fact, the muscle protein synthesis rate is saturable and reaches a plateau at ~30 g of proteins; any excess is degraded and oxidized [178,179]. Furthermore, basal values for the protein synthesis rate are restored after ~2.5 h, even though amino acid concentration in plasma is still elevated [180,181]. 

Ultimately, several studies have described a relationship between a protein-rich diet and an increased risk of metabolic diseases in healthy people. Protein intake stimulates insulin and glucagon secretion in a dose-dependent manner, contributing to glucose homeostasis [182,183]. While, in people with obesity or type 2 diabetes, the insulinotropic effect of proteins can help control hyperglycaemia [184,185,186], in healthy subjects, the same effect could be the cause of insulin resistance, most likely through the downregulation of the insulin receptor or causing post-receptor defects in the insulin signalling pathway [187,188,189]. However, some studies linked the development of metabolic deregulation to the consumption of animal proteins [190,191,192], while vegetable proteins were linked to a 25% decreased risk of type 2 diabetes [193]. Either insufficient or excessive protein intake can have adverse health consequences. Inadequate consumption may lead to amino acid deficiencies and muscle wasting. Protein overconsumption may lead to metabolic perturbations. Based on the values proposed by the Institute of Medicine (IOM), the recommended daily protein intake (RDI) to prevent loss of body nitrogen (muscle wasting) is 0.8 g/kg per day for normally active adults, 1.2 to 1.4 g/kg per day for endurance athletes and 1.6 to 1.8 g/kg per day for individuals involved in resistance or speed training [194]. 

When training, fat intake is also important. However, fat requirements are similar among athletes and slightly higher than non-athletes, and should represent the 20%–35% of total daily calories [195].

Micronutrients, which means vitamins and minerals, assist macronutrients in providing health benefits, even though their ergogenic effect (enhancing physical performance) is still controversial. An active debate still exists on the effect of antioxidants as a means to protect muscle fibers from oxidative damage caused by exercise. Vitamin E supplementation reduces oxidative stress and exercise-induced lipid peroxidation (measured by increased amounts of exhaled pentane), as well as circulating aspartate transaminase and β-glucuronidase [37,196]. Although vitamin E is a powerful antioxidant, the interaction of vitamin E with a free radical causes the formation of a vitamin E radical, with a decrease in vitamin E functionality. Vitamin C can recycle vitamin E back to its reduced state, but vitamin C concentration should remain under a certain limit (~1 mmol × 1^−1^) in order to avoid acquiring prooxidant effects, in the presence of transition metals in particular [197]. In an experimental study, four-week vitamin C (2 × 500 mg/day) and E (400 IU/day) supplementation did not reduce skeletal muscle oxidative stress or enhance mitochondrial biogenesis upon acute exercise (10 × 4 min cycling at 90% VO_2_ peak, 2 min active recovery) [198]. Recently, a growing interest has been conveyed towards the effects of antioxidant polyphenols in the modulation of physical performance and oxidative stress prevention. Flavonoids, including flavones, isoflavones, flavanones, anthocyanins and catechins, exert antioxidant activity through the inhibition of inflammatory enzymes (e.g., lipoxygenase, cyclooxygenase, xanthine oxidase, NADH-oxidase, phospholipase A2) or the quenching of peroxyl, hydroxyl, superoxide radicals and hydrogen peroxide [199]. Catechins, highly abundant in black and green tea and red wine, were also reported to prevent the radical-mediated depletion of vitamin E [200]. Polyphenols appear to have several molecular targets, leading to the activation of distinct signaling pathways, like those mediated by NF-κB, SIRT1, MAPK’s, and heat shock proteins (HSP). Their antioxidant properties rely also on the overexpression of protective inducible genes involved in the cellular stress response, such as those activated by the nuclear factor E2-related factor 2 (Nrf2) [201]. The supplementation of several antioxidants, including curcumin (known also as diferuloylmethane, the main phenolic compound from turmeric), has been considered a potential strategy for preventing oxidative stress and improving sport performance [202]. Reduced post-exercise inflammation was associated with curcumin supplementation, probably via the inhibition of NF-κB and activator protein-1 (AP-1), responsible for the expression of oxidative enzymes (cyclooxygenase-2 and 5-lipoxygenase), cytokines (TNF-α) and proinflammatory interleukins (IL-1, IL-6, IL-8), important activators of the immune system [203,204,205]. Other benefits of curcumin supplementation were the reduction of muscle damage (due to decreased plasma creatine kinase levels) [205], sport performance enhancement [206,207], and the increase in strength and muscle repair (through the modulation of NF-κB) [208]. 

Several minerals seem to exert an ergogenic effect. In particular, sodium phosphate increases maximal oxygen uptake and endurance capacity; sodium chloride maintains fluid and electrolyte balance; zinc decreases exercise-induced changes in immune cells [209]. 

However, there are currently no clear guidelines about micronutrient supplementation to improve exercise performance. According to the ACSM, in the presence of a healthy and diversified diet, vitamin and mineral supplementation is not required, but it may be prescribed to vegans and vegetarians (vitamin B12, iron, calcium, vitamin D, riboflavin and zinc supplementation) or athletes recovering from injuries [152]. 

To conclude, to date, it is still an ambitious purpose to create comprehensive and non-conflicting guidelines about macro- and micro-nutrient requirements to support exercise training, as individual requirements strongly vary, in dependence on individual features and the physical activity performed. Moreover, nutrition not only has the task of providing energy and the enzymatic cofactors necessary to carry out an optimal exercise performance, but it can also endorse the previously mentioned molecular pathways, as described below.

### 4.2. Nutrition to Regulate Inflammation and Control Redox Stress

Recently, inflammation has been addressed as an important causative factor for the major chronic diseases of modern industrialized societies, like cardiovascular disease, metabolic and neurodegenerative disorders and cancer. Prolonged low-grade inflammation is linked to an excess of oxidative stress and an unbalanced glucose and lipid metabolism in adipose, muscle and hepatic tissues [210]. It has been widely demonstrated that diet has a key role in modifying the physiological balance towards health (fighting inflammation) or disease (promoting inflammation). Foods with high glycemic index (GI) and glycemic load (GL) have been linked to an enhanced risk of coronary heart disease and type 2 diabetes mellitus, especially among overweight individuals [211]. A high glycemic index diet has been associated with increased C-reactive protein (CRP), although with some inconsistency in the results [211,212,213]. However, increasing fiber content seems to be beneficial in reducing inflammation. High fiber content was inversely correlated with CRP levels (≥3.3 g/MJ fiber gave 25–54% CRP reduction) [214], most likely associated with the reduction of fasting glucose [215]. 24 g/day fiber dose was also able to lower the levels of other important inflammation mediators, such as interleukin-6 (IL-6) and tumor necrosis factor-alpha (TNF-α) [216]. 

The quantity and quality of fat introduced with the diet can modulate the inflammatory response by enhancing the expression of pro-inflammatory adipose tissue genes [217]. The National Health and Nutrition Examination Survey (NHANES 99-00) found that high levels of saturated fatty acids (SFA) in serum phospholipids led to increased inflammatory markers, including CRP and fibrinogen. Moreover, TNF-α was positively affected by SFA in patients with heart failure [218]. Trans-fatty acid (TFA) intake was associated with increased levels of IL-6, CRP [219] and TNF-α [218]. Some studies showed that monounsaturated fatty acids (MUFA) consumption promotes anti-inflammatory gene expression [220]. Among polyunsaturated fatty acids (PUFA), dietary consumption of omega-3 (ω-3) fatty acids was linked to a decrease in inflammation markers IL-6, matrix metalloproteinase 3 (MMP-3) [221] and CRP [222]. PUFA omega-6 (ω-6) fatty acids instead, showed both pro- and anti-inflammatory effects [223]. However, recent studies showed that more than ω-3 and ω-6 alone, their ratio is what matters. In fact, an unbalanced ω-6/ω-3 ratio (in favor of ω-6) highly promotes inflammation, contributing to the onset of atherosclerosis, obesity, and diabetes [224,225]. Over the last 100–150 years, dietary patterns significantly changed, with an increase in the consumption of ω-6 fatty acids at a detriment of ω-3 fatty acids. This has led to an imbalance in the ω-6/ω-3 ratio, which is now very different from the original 1:1 ratio observed in the past [226]. Thus, the consumption of food rich in ω-3 fatty acids, like salmon, mackerel, anchovies, walnuts, flaxseed or seaweed in the context of a balanced diet, is highly recommended to restore a proper ω-6/ω-3 ratio and reduce inflammation. At this regard, many studies have looked to the Mediterranean diet and to its anti-inflammatory potentials [227]. The Mediterranean diet, typical of the southern Europe regions, is in fact characterized by a high consumption of monounsaturated olive oil (as a major source of fat), ample consumption of vegetables, fruits, legumes, cereals, and fish, with a moderate intake of red wine (source of the antioxidant polyphenols, mainly resveratrol) [228]. It follows that this diet pattern is high in α-linolenic acid (ω-3) and low in linoleic acid (ω-6), with an ω-3/ω-6 ratio of 1:7 [229]. It has been found that a higher adherence to the Mediterranean diet led to lower levels of inflammation and/or oxidative stress markers, such as plasmatic CRP, IL-6, TNF-α and nitrotyrosine levels [230]. Since oxidative stress and inflammation have been linked also to enhanced telomere shortening [231], the adherence to the Mediterranean diet can help to preserve telomere length by reducing overall inflammation. Thus, the anti-oxidant and anti-inflammatory properties of a healthy diet, in combination with proper physical activity levels, could be extremely helpful in delaying the aging process, postponing all pathologies related to increasing age.

A question that can arise is whether a diet rich in antioxidants or antioxidant supplementations may be useful to counteract exercise-induced oxidative stress during physical activity. The high demand of ATP from the training skeletal muscles, translates into a massive release of ROS by active mitochondria. An antioxidant-rich diet could work in synergy with endogenous defenses and may be a tool for preventing or reducing exercise-induced oxidative stress. However, an excess of exogenous antioxidants may have a negative impact on physical performance. Indeed, basal ROS levels act as regulatory mediators in maintaining redox balance. Thus, high doses of antioxidants may compromise the redox homeostasis and even shift towards pro-oxidant effects. From several studies, it emerged that exogenous antioxidants can suppress the muscle adaptive response to exercise-induced oxidative damage. Experimental trials proved that vitamin C supplementation was able to suppress the heat shock protein (HSP) response in lymphocytes to hydrogen peroxide [232], but the same effect wasn’t achieved upon vitamin E and β-carotene supplementation, which instead resulted in increased HSP70 and HSC70 muscle content, even though the effect was no longer seen after 8 weeks of supplementation [233]. Ristow et al., found that molecular mediators of ROS defense were induced upon exercise, but their levels dropped upon antioxidant supplementation (vitamin C and vitamin E) [234]. From a dietary perspective, a varied and balanced diet, rich in fruits, fibers and vegetables, offering the correct ratios of vitamins, minerals and antioxidants, could be useful to support exercise-induced oxidative balance [235]. Apparently, like a double-edged sword, physical activity can help to fight oxidative stress when practiced with moderation (and, in this case, an excessively antioxidant-rich diet or antioxidant supplementations would be deleterious, nullifying the positive effects of exercise). On the other hand, when exercise is too strenuous, oxidative stress and cell damage would exceed its beneficial effects. In this instance (and in all cases of physiological deficiencies), an exogenous source (diet and/or supplements) of antioxidants might be useful to limit the oxidative damage. In any case, a healthy and balanced diet is definitely recommended since it is proven to be essential to support any kind of physical activity and, together, these two are key to achieving both physiological and mental well-being.

### 4.3. Nutrition to Support Epigenetic Regulations

Among environmental factors, nutrition has a big impact on human health and longevity, having the power to predispose to or protect from the onset of many pathologies, including metabolic disorders, cardiovascular diseases, neurodegeneration and cancer. Increasing evidence suggests that diet can epigenetically influence gene expression [236]. In this regard, nutritional epigenetics has gained popularity as a promising tool to prevent and delay aging-associated processes and diseases [237].

Plenty of food components can modify the expression of critical genes associated with physiological and pathological processes, by modulating the function of epigenetic enzymes, such as DNA methyltransferases (DNMT), histone deacetylases (HDAC) or histone acetyltransferases (HAT), or altering their substrates’ availability [238]. Many epidemiological studies have linked adverse environmental factors [239] and impaired nutrition during gestation and early life [240,241] to epigenetic dysregulation, with an increased risk for developing several diseases later in adulthood. Adverse maternal nutrition in the periconceptional period has been linked to compromised postnatal health with enhanced disease risks [242]. One of the best known examples of the effects of starvation during pregnancy is the Dutch Famine, the consequence of a German-imposed food embargo in the western part of The Netherlands in the winter of 1944–45. Caloric restriction was associated with altered methylation patterns to specific imprinted genes (such as the insulin growth factor 2, IGF-2), which led to increased risk for type II diabetes, cardiovascular disease, metabolic disorders and decreased cognitive function in the offspring and the F2 generation [243], highlighting the transgenerational effect of a poor maternal diet (and paternal as well) [244]. The famine suggested that maternal nutrient deficiency early in gestation can lead to various adverse metabolic or mental phenotypes.

In particular, maternal methyl-donor nutrient deficiency during the periconceptional period can modulate the DNA methylation patterns in offspring with negative consequences for adult health-related phenotypes. Folate, a water-soluble B vitamin, and its derivatives, have been extensively studied for their effect on DNA methylation. Folates support vital physiological processes, like cell division and homeostasis, working as methyl donors in the one-carbon metabolism, a complex network of reactions involved in biosynthetic pathways (conversion of serine to glycine, histidine catabolism and synthesis of thymidylate, methionine, and purine), in which one-carbon units are transferred through different substrates up to the universal methyl donor: the *S*-adenosyl-l-methionine [245]. Folate metabolism is a delicate mechanism, which, besides genetic polymorphism, is influenced by folate dietary intake [246]. Low dietary folate intake is a serious concern during gestation because it has been associated with aberrant DNA methylation profiles and increased risk of neural tube defects in the foetus. Moreover, periconceptional folic acid supplementation (400 mg/day) enhances the methylation of the insulin-like growth factor 2 gene (IGF-2), an imprinted gene, whose expression is repressed when methylated [247].

Moreover, other methyl-donor nutrients such as (methionine, choline, betaine, and vitamin B12) can influence the DNA methylation status. In an animal study, folate, vitamin B12 and methionine deficiencies in the periconceptional period altered the methylation status of 4% of 1400 CpG islands, causing obesity and altered immune responses in adult offspring [248].

5-methyl-THF, the most reduced form of folate, is produced by the cytosolic and widespread NADPH-dependent methylene tetrahydrofolate reductase (MTHFR) and it is essential for the remethylation of homocysteine to methionine, via the methionine synthase enzyme, that uses cobalamin (vitamin B12) as a cofactor. In mammalian cells, methionine can also be regenerated from homocysteine, thus in a folate-independent manner, using betaine (a product of choline degradation) in a reaction catalysed by the betaine-homocysteine methyltransferase (BHMT), which is expressed in the liver and kidneys [249]. Homocysteine remethylation is very important because, not only is homocysteine cytotoxic and high levels are linked to cardiovascular disease [250], but also because methionine is the substrate for *S*-adenosylmethionine (SAM) synthetase. SAM is the universal methyl-carrier, thus it plays a major role in epigenetics and in many biosynthetic processes, including creatine, polyamine and phosphatidylcholine synthesis [251,252,253]. In particular, phosphatidylcholine synthesis represents the largest source of *S*-adenosylhomocysteine (SAH) [254]. Changes in methionine concentration alter the SAM/SAH ratio, with consequences on many methylation reactions, including DNA and histone methylation [255].

Not only specific micronutrients can affect epigenetic regulations, but also fasting and caloric restriction that, without malnutrition, were proved to be beneficial in increasing lifespan [256]. The positive effects of caloric restriction seem to be mediated by reduced oxidative stress, increased DNA repair, downregulation of pathways that lead to apoptosis and cell senescence, and increased mitochondrial function. A calorie restriction regimen favours the inhibition of critical inflammatory genes, such as NF-κB [238], through the activation of specific cellular signalling pathways, among which are those mediated by sirtuins, which are NAD^+^-dependent histone deacetylases (HDACs). Some dietary components (for example resveratrol) can activate sirtuin 1, which has been proven to be a means by which caloric restriction can delay or reverse some of the physiological changes associated with aging [257].

Since inhibition of HDACs could derepress epigenetically silenced genes, many studies have focused on whether certain bioactive food components can act as modulators of histone deacetylases (HDAC) and histone acetyltransferases (HAT). In this regard, butyrate, a short-chain fatty acid from dietray fiber, sulforaphane, an isothiocyanate found in broccoli sprouts, or diallyl disulfide, an organosulfur compound present in garlic, have anti-carcinogenic potential, by inhibiting HDACs or enhancing HATs [258,259,260,261,262]. Indeed, numerous bioactive compounds contained in food mediate epigenetic processes in our body. Natural foods, like fruits, vegetables and spices, have proven to potentially prevent or treat a range of pathologies, thanks to the bioactive compounds they are rich in. These latter compounds can elicit protective effects through complex molecular mechanisms, including epigenetic DNA methylation and histone modifications. A number of bioactive molecules with a key role in controlling expression of genes involved in cell proliferation, death and differentiation are known, including polyphenols (genistein, curcumin, resveratrol, apigenin and gingerol), retinoids, essential fatty acids, isothiocyanates, sulforaphane, silymarin, diallyl sulfide, lycopene, and rosmarinic acid. These bioactive components exert anti-cancer potentials through the modulation of epigenetic targets. For example, epigallocatechin-3-gallate, the main catechin from green tea, through the inhibition of DNMTs, allows the expression of tumor suppressor genes, including p16, retinoic acid receptor beta (RARβ), glutathione *S*-transferase pi (GSTP) and methylguanine-DNA methyltransferase (MGMT) in several human cancer cell lines [263]. Flavonoids, vitamins and carotenoids are also protective agents against metabolic syndromes, such as diabetes and coronary disease [264,265]. Isothiocyanates from cruciferous vegetables, allyl sulfides from garlic, retinoids, and ω-3 fatty acids mainly modulate HDACs activity [266]. Butyrate, a short-chain fatty acid produced by the bacterial fermentation of fibers in the colon, potentially inhibits HDACs activity, resulting in transcriptional regulation [267]. In this complex picture, there also emerges the role of another important player: the gut microbiome. The microbiota, which exists in symbiosis with mammalian organisms, consists of approximately 1014 microorganisms, including bacteria, archaea and viruses, that colonize the intestinal mucosal surfaces [268] and exert important functions, from the digestion of food to the regulation of the immune system [269]. Interestingly, gut microorganisms synthesize numerous bioactive compounds, like short chain fatty acids (predominately acetate, butyrate, and propionate), choline metabolites and lipids [269], which are beneficial to the host. The effects the microbiota-derived metabolites exert are not only local, but can also target distant organs, such as the liver, heart and the central nervous system [270,271]. Not surprisingly, intestinal dysbiosis (alterations in the composition and function of the gut microbiota) is a causative factor of several diseases, such as obesity, diabetes, metabolic syndrome and colorectal cancer [272,273,274], mainly via epigenetic changes (DNA methylation, histone modifications and regulation by noncoding RNAs) induced by microbial metabolites, including short-chain fatty acids, folates, biotin and trimethylamine-*N*-oxide [275,276]. Diet is a key factor in the establishment of the gut microbiota composition and in the shaping of the host’s metabolism [271,277]. A dietary pattern high in fats and carbohydrates, how the “Western” diet can be, alters microbial communities’ composition and predisposes to obesity [278]. In contrast, plant-based diets or diets rich in certain bioactive molecules (such as gingerol), favour the proliferation of specific bacteria strains that protect from obesity [279].

An unbalanced diet can also result in aberrant miRNA expression. A methyl- and folate-deficient diet can induce prominent early changes in expression of microRNA genes, including miR-34a, miR-127, miR-200b, and miR-16a involved in the regulation of apoptosis, cell proliferation and epithelial-mesenchymal transition [280]. Certain dietary components, such as curcumin and retinoic acid, may protect against severe disease, such as cancer, through miRNA regulation [238].

As a consequence of all these aspects, not only individual nutrients, but the whole dietary pattern affects epigenetic regulations implied in plenty of different pathways in the body. In the Western diet, for example, saturated fats, red meats, and refined carbohydrates tend to exceed fresh fruits and vegetables, whole grains, seafood, and poultry. Not surprisingly, this dietary regimen has been linked to hypertension, heart disease, diabetes and increased risk of cancer, outcomes that likely occur via epigenetic alterations [281,282,283,284]. By contrast, the Mediterranean diet, which is traditionally rich in fruits, vegetables, fish and poultry, whole grains and healthy fats (monounsaturated and polyunsaturated fats), but poor in red meat and saturated fats, is associated with a reduced risk of overall mortality [285,286]. An antioxidant-rich diet is highly anti-inflammatory, fosters cellular antioxidant defenses and prevents those associated with low-grade systemic inflammation. Antioxidant-rich foods avoid ROS accumulation (even in response to intense physical exercise) and prevent their interaction with DNA, proteins, lipids, and carbohydrates, which lead to an alteration of the cellular metabolic activity and eventually induce a diseased phenotype. Moreover, ROS can directly modulate the epigenome, since epigenetic regulations are strictly connected to the metabolic state of the cell.

First of all, ROS inhibits methionine synthase activity. This is necessary so that the cell stimulates the diversion of homocysteine to produce the antioxidant glutathione, but it also affects the bioavailability of the universal methyl-donor, SAM [287]. Moreover, ROS-induced hydroxylation of pyrimidines and 5-methylcytosine (5mC) can interfere with epigenetic signals related to 5-hmC (due to structural similarities) [288], while TET-mediated hydroxymethylation (which depends on NAD^+^ and α-ketoglutarate levels) can regulate DNA demethylation [289]. Similarly, cellular levels of Acetyl-CoA, NAD^+^ and *S*-adenosylmethionine modulate the activity of histone-modifying enzymes, indicating that the global metabolism and energy levels of the cell are tightly linked to its epigenetic status [290].

In conclusion, nutrients and bioactive food components have a big impact on the phenotype, by reversibly altering the epigenetic imprint and, subsequently, influencing gene expression. Thus, diet, with the power to push towards health or disease, represents much more than merely nurture for the body.

## 5. Conclusions

Gene expression and associated epigenetic modifications represent a major factor in the regulation of the body’s response to environmental stimuli, such as physical activity. Activation of different molecular pathways in the skeletal muscles and in other tissues implied in the response to physical activity is essential for an optimized performance. The skeletal muscle, indeed, has an epigenetic memory that regulates its ability to respond to external stimuli. In this context, nutrition is not simply a source of energy for the body, but it represents a rich mixture of heterogeneous molecules, including natural antioxidants, that fosters molecular regulations, directly and through epigenetic changes. In the light of this evidence, nutri(epi)genomics represents a key factor to be considered by health practitioners, not only to promote long term health, but also to endorse optimized physical performances.

## Abbreviations

5mC	5-methylcytosine
ACSM	American College of Sports Medicine
ADP	Adenosine diphosphate
ALA	Delta-aminolevulinic acid
AMP	Adenosine monophosphate
AMPK	AMP-activated protein kinase
ASC	Apoptosis-associated speck-like protein containing a CARD
ATP	Adenosine triphosphate
BDNF	Brain-derived neutrophic factor
BHMT	Betaine-homocysteine methyltransferase
BW	Body weight
cAMP	Cyclic adenosine monophosphate
CRP	C reactive protein
Cyt c	Cytochrome c
DNMT	DNA methyltransferases
	
EAA	Essential amino acids
ecNOS	Endothelial nitric oxide synthase
GI	Glycemic index
GL	Glycemic load
GLUT-4	Glucose transporter 4
GSTP	Glutathione *S*-transferase pi
HAT	Histone acetyltransferases
HDAC	Histone deacetylases
HSPs	Heat shock proteins
IGF-1	Increased insulin-growth factor-1
IGF-2	Insulin growth factor-2
IL-1,6,10	Interleukine 1, 6, 10
IL-1ra	Interleukin-1 receptor antagonist
MAPKs	Mitogen-activated protein kinases
MEF2A	Myocyte enhancer transcription factor 2A
MEF2D	Myocyte enhancer transcription factor 2D
MGMT	Methylguanine-DNA methyltransferase
miRNAs	microRNAs
MMP-3	Matrix metalloproteinase 3
mTFA	Mitochondrial transcription factor
MTHFR	Methylene tetrahydrofolate reductase
MUFA	Monounsaturated fatty acids
NAD^+^	Oxidized nicotinamide adenine dinucleotide
NADPH	Reduced nicotinamide adenine dinucleotide phosphate
NF-κB	Nuclear factor kappa-light-chain-enhancer of activated B cells
NHANES	National Health and Nutrition Examination Survey
NK	Natural killer cells
NO	Nitric oxide
NRF-1	Nuclear respiratory factor 1
PGC-1α	Peroxisome proliferator-activated receptor gamma coactivator 1-alpha
PKA	Protein kinase A
PPARD	Peroxisome proliferator-activated receptor delta
PPARγ	Peroxisome proliferator activated receptor gamma
PUFA	Polyunsaturated fatty acids
RARβ	Retinoic acid receptor beta
RNS	Reactive nitrogen species
ROS	Reactive oxygen species
SAH	*S*-adenosylhomocysteine
SAM	*S*-adenosylmethionine
SFA	Saturated fatty acids
SOD	Superoxide dismutase
TFA	Trans-fatty acid
TNF-R	Tumor necrosis factor receptor
TNF-α	Tumor necrosis factor alpha
vitamin B_12_	Cobalamin
WHO	World Health Organization
β-AR	Lipolysis-inducing β-adrenoceptor
ω-3	Omega-3
ω-6	Omega-6
